# Molecular characterization and pathogenicity evaluation of enterovirus G isolated from diarrheic piglets

**DOI:** 10.1128/spectrum.02643-23

**Published:** 2023-10-13

**Authors:** Yassein M. Ibrahim, Wenli Zhang, Xinrong Wang, Gebremeskel Mamu Werid, Lizhi Fu, Haidong Yu, Yue Wang

**Affiliations:** 1 College of Veterinary Medicine, Southwest University, Chongqing, China; 2 Harbin Veterinary Research Institute, Chinese Academy of Agricultural Sciences, Harbin, China; 3 Chongqing Academy of Animal Science, Chongqing, China; 4 National Center of Technology Innovation for Pigs, Chongqing, China; Changchun Veterinary Research Institute, Changchun, China

**Keywords:** enterovirus G, characterization, genetic recombination, pathogenicity

## Abstract

Four enterovirus G (EV-G) strains were isolated from diarrheic piglets that were negative for common swine enteric viruses. The spherical enterovirus particles of roughly 30-nm diameter were observed under transmission electron microscopy by using plaque-purified enterovirus. The complete genome sequence analysis revealed that each of four enteroviruses contained a papain-like cysteine protease (PLCP) gene between the 2C and 3A junction regions of the viral genome. This insertion encoded a predicted protease similar to the PLCP of porcine torovirus. The phylogenetic analysis based on complete genome with and without *PLCP* gene revealed that the four isolated EV-G strains were grouped together with global enterovirus G1-PLCP strains, and more closely related to EV-G/PLCP strains previously detected in China, Japan, and Korea (90.3%–92.2% similarities based on nucleotides). The cell susceptibility test demonstrated that the isolated EV-G could infect and replicate in cell lines from various host species. Furthermore, pathogenicity evaluation showed that the isolated EV-Gs induced mild diarrhea, pyrexia, and reduced body weight in infected piglets. The epidemiological investigation revealed a high prevalence of EV-G in swine herds. Together, our findings demonstrate that the isolated EV-G is pathogenic in piglets and may be advantageous in providing more trustworthy data on the evolution and pathological properties of EV-G.

**IMPORTANCE:**

Enterovirus G is a species of positive-sense single-stranded RNA viruses associated with several mammalian diseases. The porcine enterovirus strains isolated here were chimeric viruses with the *PLCP* gene of porcine torovirus, which grouped together with global EV-G1 strains. The isolated EV-G strain could infect various cell types from different species, suggesting its potential cross-species infection risk. Animal experiment showed the pathogenic ability of the isolated EV-G to piglets. Additionally, the EV-Gs were widely distributed in the swine herds. Our findings suggest that EV-G may have evolved a novel mechanism for broad tropism, which has important implications for disease control and prevention.

## INTRODUCTION

Enteroviruses are a group of non-enveloped positive-sense, single-stranded RNA viruses classified under the order Picornavirales and the family Picornaviridae. Enteroviruses are a large and very diversified genus of viruses that are characterized by high mutation and recombination rates (1 – 5). The genus *Enterovirus* contains viruses that infect humans (species A–D), cattle (species E and F), pigs (species G), and non-human primates (species A, B, D, H, and J) (6, 7). Viruses of the genus *Enterovirus* are small (~27 nm) in diameter, with icosahedral symmetry. The genome of an enterovirus ranges in size from 7,400 to 7,500 nucleotides (nt) and consists of a single open reading frame (ORF) flanked by 5′ and 3′ untranslated regions (UTRs) as well as a 3′ poly(A) tail. The 5′-UTR is around 700–825 nucleotides in length, which comprises secondary structural elements that are required for RNA replication as well as an internal ribosome entry site for the commencement of translation (8). The 3′-UTR of approximately 75–100 nucleotides in length contains complex cis-acting regions that are critical for RNA replication (9). The ORF encodes a single polyprotein that is subsequently cleaved by a post-translational process by virus encoding proteases into three precursor protein products (P1, P2, and P3) that are further processed into four structural proteins (VP1, VP2, VP3, and VP4) and seven non-structural viral proteins (2A, 2B, 2C, 3A, 3B, 3C, and 3D) (10, 11).

Porcine enteroviruses (PEVs) were initially classified into 13 serotypes (PEV-1 to PEV-13) based on viral neutralization test results (12, 13). Based on genomic analyses, however, PEV-1 to PEV-7 and PEV-11 to PEV-13 have been re-classified into a new genus, *Teschovirus*; PEV-8, formally named PEV‐A, has been re-classified into a new genus, *Sapelovirus* (14, 15), while PEV-9 and PEV-10, formally belonging to PEV‐B, have been re-classified as enterovirus G (EV-G) (16, 17). PEV-9 and PEV-10, the prototypical EV-Gs isolated in 1973 and 1975, were renamed as EV-G1 and EV-G2, respectively. EV-G now encloses 20 serotypes (EV-G-1 to EV-G-20) (6). EV-G has recently been found in wild boars (EV-G4 and EV-G16), sheep (EV-G5 and EV-G7), and goats (EV-G20) (18 – 22). According to reports, EV-G infections were found to be prevalent among pig herds in many countries (23 – 28).

Swine are susceptible to a wide range of enteric viral and bacterial species, causing the global pork industry substantial economic losses. In majority of pig-producing countries, diarrhea is frequently caused by single or mixed infections of enteric coronaviruses, such as porcine epidemic diarrhea virus (PEDV), transmissible gastroenteritis virus (TGEV), and porcine deltacoronavirus (PDCoV) or rotaviruses, which are frequently identified in veterinary diagnostic laboratories using a reverse transcription PCR (RT-PCR)-based approach (29, 30). In a few clinical cases, none of the above-mentioned viral pathogens were detected in diarrheic pigs, indicating that other viruses are involved in swine gastroenteritis. Since the advent of viral metagenomics (31, 32), genomic detection and viral isolation methods were used to identify that those diarrheic pigs were positive for kobuvirus, astrovirus, sapelovirus, or EV-Gs but negative for enteric coronaviruses and rotavirus (33 – 35). In addition, the co-circulation of numerous EV-G genotypes, G1, G2, and G17, have been identified in fecal samples from diarrheic swine in the USA, Belgium, Japan, South Korea, and China. In contrast, studies regarding EV-G isolation are limited, and most of the whole-genome sequences of EV-G have been obtained by next-generation sequencing (36, 37). In this study, we have reported the prevalence, characterization, and pathogenicity evaluation of swine enterovirus G.

## RESULTS

### Isolation and identification of EV-G strains

**FIG 1:**
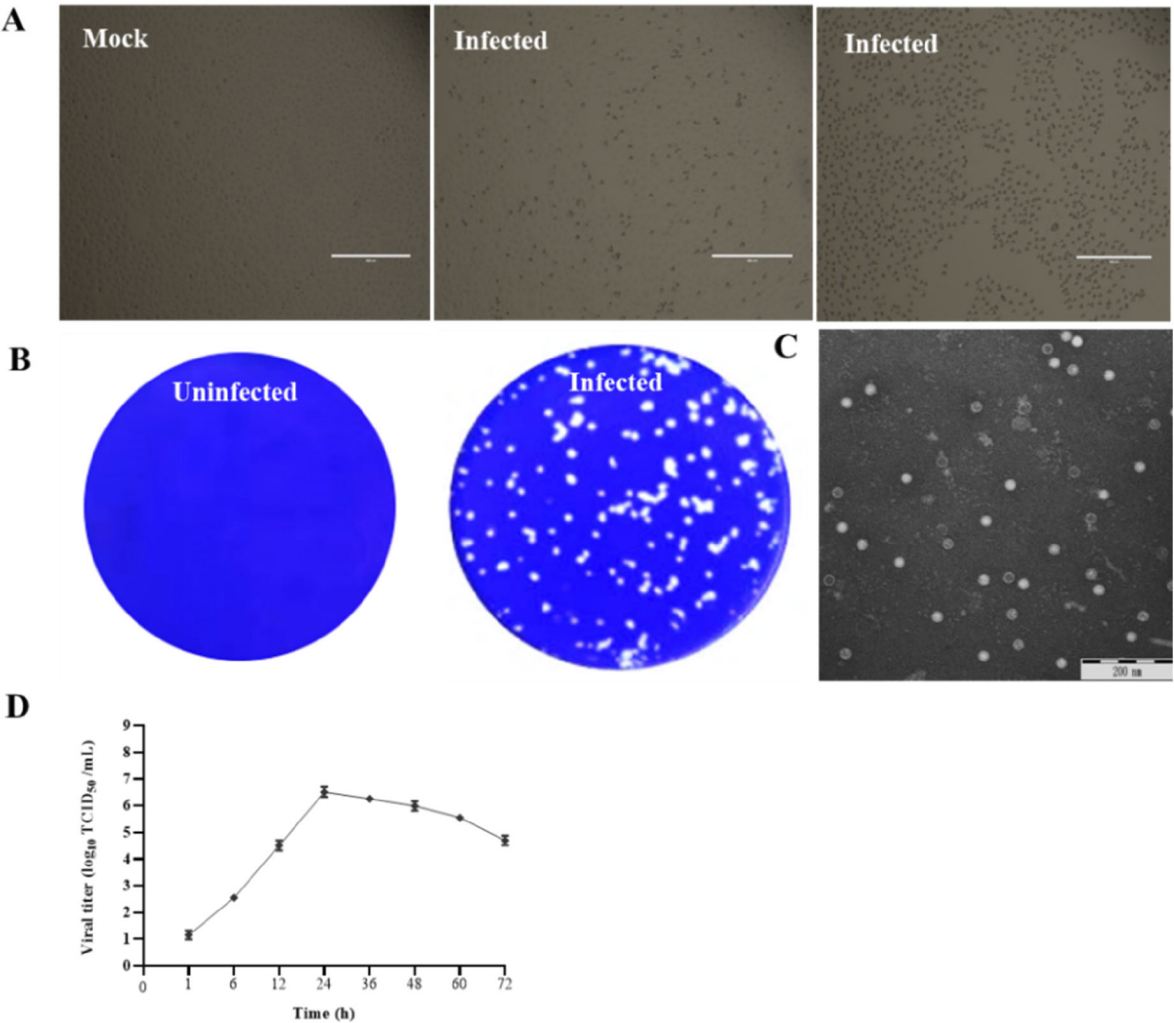
Isolation and identification of EV-G. (**A**) Cytopathic effects in EV-G-infected Marc145 cells at 24 h post-infection. (**B**) Production of plaques of the EV-G isolate in Marc145 cells. (**C**) Picornavirus-like particles under TEM. (**D**) Growth kinetic of EV-G.

Marc145 cells were inoculated with four diarrheic fecal specimens that were positive for EV-G but negative for other common enteric viruses. After three blind passages on Marc145, EV-Gs developed cytopathic effect (CPE), characterized by cell rounding, shrinking, and detaching, compared to uninfected control cells (Fig. 1A). After four rounds of plaque purification, the purified virus developed a uniform and clear plaque under an agar overlay medium (Fig. 1B). After ultracentrifugation, the plaque-purified viruses were examined under transmission electron microscopy (TEM). Spherical, non-enveloped picornavirus-like particles of approximately 25–30 nm in diameter were observed (Fig. 1C). The RT-PCR and whole-genome sequencing confirmed that the isolated viruses were EV-G. To determine the growth kinetics of the EV-G isolates, Marc145 cells were inoculated with the purified virus at an multiplicity of infection (MOI) of 0.01, and the mean titers of three independent measurements at the indicated time points were measured. The results showed that the virus replication reached a peak of 10^6.5^ 50% tissue culture infectious dose (TCID_50_)/mL at 24 h, suggesting that the multiplication cycle of the isolated virus is completed within 24 h (Fig. 1D).

### Whole-genome sequence analysis of the isolated EV-G strains

**FIG 2:**
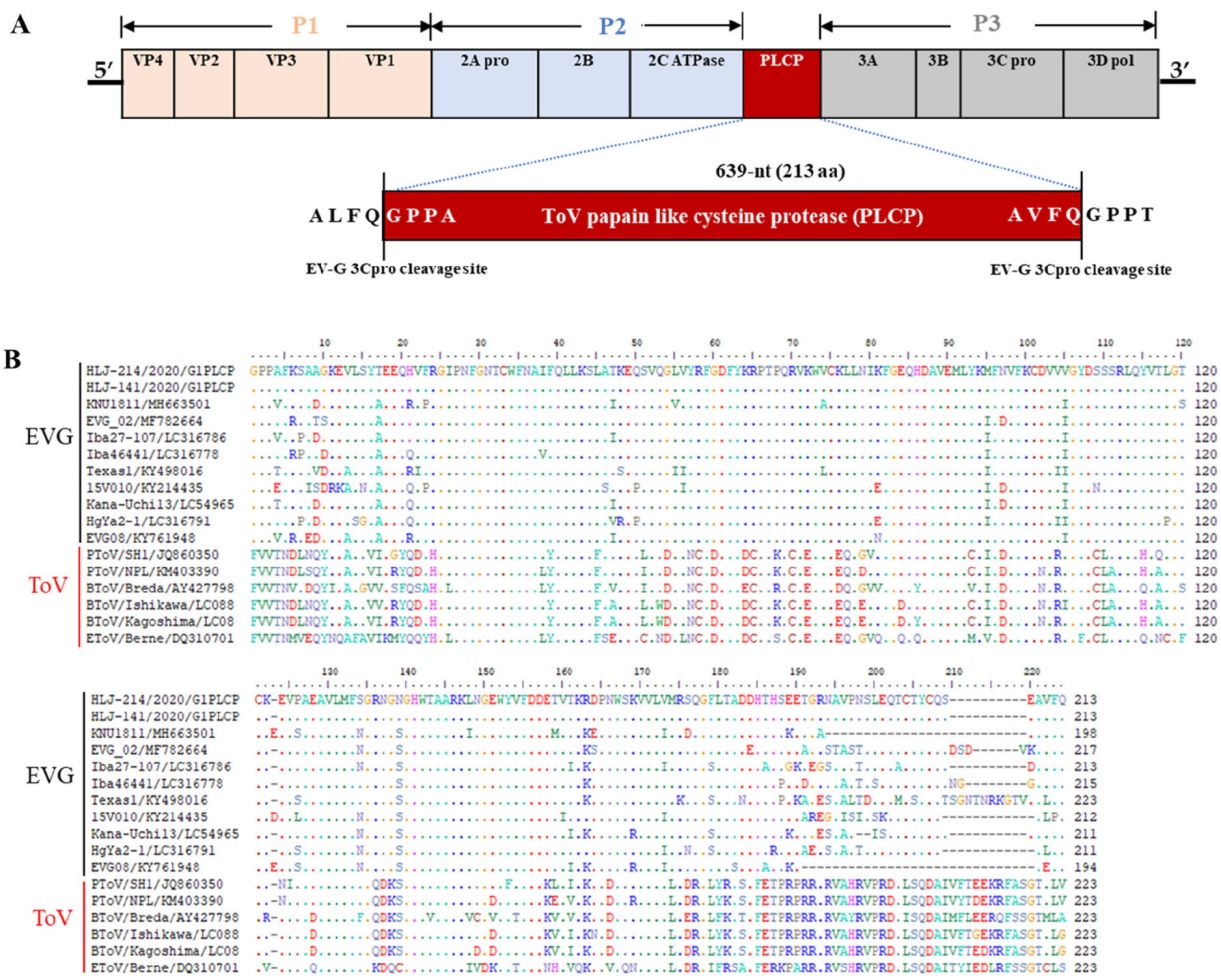
Schematic diagram of the genome organization of isolated EV-G. (**A**) The single ORF is flanked by 5′-UTR (813 nucleotides) and a short 3′-UTR (71 nucleotides), followed by a poly(A) tail. The torovirus (ToV)-*PLCP* gene is presented as a red box that locates at viral 2C/3A cleavage junction. The flanked sequences of putative 3C protease cleavage sites are shown in an enlarged red box. Vertical lines indicate the polyprotein processing site by the 3C protease. (**B**) Multiple alignment of the amino acid sequences of the PLCP regions of the recombinant EV‐G and ToV strains.

Using next-generation sequencing technology, we obtained complete genome sequences of the four EV-G isolates, which were designed as CH/HLJ-141/G1PLCP/2020, CH/HLJ-214/G1PLCP/2020, CH/HLJ-312/G1PLCP//2020, and CH/HLJ-315/G1PLCP//2020, respectively. To understand the molecular characteristics of the isolated viruses, we compared the genomes of previously identified EV-G strains in GenBank, with the genomes of these four isolated viruses. The results revealed that the entire genomes of four isolated EV-G strains, excluding poly(A) tail, are all 8030 nucleotides, flanked by 5′-UTR of 813 nucleotides and 3′-UTR of 71 nucleotides. The complete genome composed of a single ORF of 7,146 nt and encoded 2,382-amino acid (aa) polyprotein, which cleaved into P1 (VP4, VP2, VP3, and VP1), P2 (2A, 2B, and 2C), and P3 (3A, 3B, 3C, and 3D) with 2505 nt/835 aa, 1734 nt/578 aa, and 2268 nt/756 aa, respectively, as well as an exogenous papain-like cysteine protease (PLCP) of 639 nt/213 aa at the 2C/3A junction region (Fig. 2; Table S1). Furthermore, identity comparisons of complete genomes demonstrated that EV-Gs identified in this study had 70.1%–92.2% similarities in nucleotide and 80.9%–97.4% similarities in amino acid, compared to the EV-G reference strains available in the GenBank database (Table 1). The entire genome of isolated EV-G strains shared 78.1–80.4% nucleotide similarities and 88.3%–89% amino acid similarities, respectively, with the prototype EV-G1 strains (GXQZ/China, Chahf1/China, Iba46431/Japan, 1303212/USA, and UKG/410/73). However, the isolated EV-G strains showed relatively higher sequence homology (86%–92.2% nucleotide similarities and 94.7%–97.4% amino acid similarities at the genome level) with the chimeric EV-G1 strains from China, Korea, Japan, US, and Belgium but exhibited lower homology (81.6%–81.7% nucleotide similarities and 87.8%–88.5% amino acid similarities) with the chimeric EV-G2 and EV-G17 strains, respectively.

**TABLE 1:** Homology comparison of the whole genome of the isolated EV-Gs with reference strains^*a*^

No.	Strain	Year	Host	Country	Accession no.	Whole-genome sequence identity (%)	
						nt	aa
1	GXQZ/G1	2017	Swine	China	MT274669	78.1	88.3
2	Iba46431/G1	2015	Swine	Japan	LC316790	79.6	89.4
3	1303212 /G1	2013	Swine	USA	KF985175	78.6	87.3
4	Chahf1/G1	2008	Swine	China	HM131607	79.5	89.0
5	UKG/410/73 /G1	2002	Swine	UK	AF363453	80.4	89.0
6	UKG/410/73 /G1	2006	Swine	UK	Y14459	79.7	88.0
7	EVG02/CHI/G1PLP	2014	Swine	China	MF782664	92.2	97.4
8	HgOg23/G1/PLCP	2015	Swine	Japan	LC316775	88.5	96.5
9	Iba46441/G1/PLCP	2015	Swine	Japan	LC316778	90.4	96.9
10	Kana-Uchi13/G1-PLCP	2019	Swine	Japan	LC549657	89.5	96.9
11	Iba27-107G1-PLCP	2015	Swine	Japan	LC316786	91.0	97.0
12	KNU1811/G1/PLCP	2018	Swine	Korea	MH663501	90.7	97.0
13	15V010/G1/PLCP	2015	Swine	Belgium	KY214435	86.0	94.7
14	Texas1/G1/PLpro	2014	Swine	USA	KY498016	86.3	95.3
15	Texas2/G1/PLpro	2014	Swine	USA	KY498017	86.4	95.3
16	HgYa21/G2/PLCP	2015	Swine	Japan	LC316791	81.6	88.5
17	Iba26506/G2	2014	Swine	Japan	LC316792	80.6	86.9
18	LP_54 /G2	2001	Swine	Germany	AF363455	79.1	87.0
19	Bu65/G3	2014	Swine	Japan	LC316805	82.2	89.6
20	Bu82/G3	2014	Swine	Japan	LC316806	82.1	90.2
21	Bu84/G3	2014	Swine	Japan	LC316807	71.3	89.9
22	IshiKa7/G3	2016	Swine	Japan	LC316815	76.2	83.7
23	IshiSa5/G3	2015	Swine	Japan	LC316808	77.2	84.4
24	Ishi9/G3	2019	Swine	Japan	LC535395	70.1	82.3
25	K23/G3	2008	Swine	Hungary	HQ702854	76.1	83.6
26	WBD/G4	2011	Swine	Hungary	JN807387	77.3	84.8
27	HgYa11/G4	2016	Swine	Japan	LC316818	–	–
28	TB4OEV/G5	2009	Ovine	Hungary	JQ277724	71.3	81.0
29	PEVBKOR/G6	2009	Swine	Korea	JQ818253	78.3	84.7
30	990/UKNI/G7	2018	Swine	UK	MG958646	70.3	80.3
31	724118 /G8	2012	Swine	Vietnam	KJ156437	–	–
32	714418/CaoLanh/G8	2012	Swine	Vietnam	KT265911	78.7	86.0
33	HgTa222/G9	2015	Swine	Japan	LC316821	81.7	89.8
34	Iba2720/G9	2015	Swine	Japan	LC316824	80.3	87.5
35	IshiYa32/G9	2016	Swine	Japan	LC316825	81.6	89.1
36	734087/ThanhBinh/G9	2012	Swine	Vietnam	KT265961	79.3	87.1
37	724162 /G9	2012	Swine	Vietnam	KJ156438	–	–
38	714171/CaoLanh/G9	2012	Swine	Vietnam	KT265894	79.0	87.1
39	714152/CaoLanh/G9	2012	Swine	Vietnam	KT265893	78.8	87.1
40	HgYa231/G10	2015	Swine	Japan	LC316827	79.6	88.7
41	HgYa241/G10	2015	Swine	Japan	LC316828	79.6	88.7
42	IshiIm8/G10	2016	Swine	Japan	LC316830	82.1	89.7
43	IshiKa32/G10	2015	Swine	Japan	LC316829	80.5	89.3
44	734123 /G10	2012	Swine	Vietnam	KJ156446	–	–
45	PoEnVBEL12R021/G10	2015	Swine	Belgium	KJ156451	–	–
46	744257 /G11	2012	Swine	Vietnam	KP982873	77.5	83.1
47	714222/CaoLan/G12	2012	Swine	Vietnam	KT265900	–	–
48	714270/CaoLanh/G13	2012	Swine	Vietnam	KT265903	–	–
49	714405/CaoLanh/G14	2012	Swine	Vietnam	KT265909	–	–
50	724307/ChauThanh/G15	2012	Swine	Vietnam	KT265941	–	–
51	BS14173H2/DakLak/G16	2014	Swine	Vietnam	KT266010	–	–
52	EVG08/G17PLCP	2015	Swine	USA	KY761948	81.7	87.8
53	F262/G18	2013	Swine	Germany	MF113370	–	–
54	F82/G19	2013	Swine	Germany	MF113372	–	–
55	JL14/G20	2014	Goat	China	KU297674	71.7	80.9

^*a*^ nt, nucleotide sequence identity; WGS, whole-genome sequence; –, not available.

The PLCP of isolated EV‐Gs shared 78.7%–88.6% and 80.8%–92.5% similarities in nucleotide and amino acid sequences with other recombinant EV‐G1, EV‐G2, and EV‐G17 strains from the USA, Belgium, Japan, South Korea, and China (26, 38 – 41), exhibiting the highest amino acid identity at 92.5% with the EV-G1 strain Iba46441/Japan/2015 (Table 2). Interestingly, in this study, all original fecal samples had a 639-nt insertion of porcine torovirus (ToV)-PLCP, as confirmed by RT-PCR and sequencing results (Fig. S1a). Furthermore, PLCP insertion was detected in Marc145 cells infected with the isolated EV-Gs by RT-PCR at passages 1 and 7 but not at passage 10 (Fig. S1b and c). These findings suggest that the 639-nt PLCP insertion is not stable and may be lost when the virus adapts to the host cells. Furthermore, the results revealed that recombinant EV-G strains from different geographical areas had varying lengths of the inserted PLCP (194/223aa, 212aa, 211/217aa, 198-aa, and 217-aa for the US, Belgium, Japanese, Korea, and China strains, respectively) (Fig. 2B).

**TABLE 2:** Comparison of PLCP of the isolated strains with chimeric EV-G and ToV reference strains

No.	Strain	Year	Host	Country	Accession no.	PLCP (sequence identity %)	
						nt	aa
1	EVG02/NC_CHI//G1/PLCP	2014	Swine	China	MF782664	88.0	89.7
2	Iba46441/G1/PLCP	2015	Swine	Japan	LC316778	86.2	92.5
3	Iba27-107/G1/PLCP	2015	Swine	Japan	LC316786	88.6	90.2
4	Kana-Uchi13/G1/PLCP	2019	Swine	Japan	LC549657	87.9	90.2
5	KNU1811/G1/PLCP	2018	Swine	Korea	MH663501	87.8	83.0
6	15V010/G1/PLPC	2015	Swine	Belgium	KY214435	79.1	83.0
7	Texas1/G1/PLpro	2014	Swine	USA	KY498016	83.1	80.8
8	HgYa21/G2/PLCP	2015	Swine	Japan	LC316791	85.6	87.1
9	EVG08/G17/PLCP	2015	Swine	USA	KY761948	78.7	81.2
10	PToV/SH1	2010	Swine	China	JQ860350	65.8	56.2
11	PToV/NPL	2014	Swine	USA	KM403390	65.4	55.4
12	BToV/Breda1	1998	Bovine	Canada	AY427798	60.2	49.1
13	BToV/Ishikawa	2010	Bovine	Japan	LC088094	62.9	54.0
14	BToV/Kagoshima	2014	Bovine	Japan	LC088095	63.1	54.0
15	EToV/Berne	2005	Equine	Switzerland	DQ310701	56.4	44.2

Additionally, the 5′-UTR, VP4, VP3, VP2, VP1, 2A, 2B, 2C, 3A, 3B, 3C, 3D, and 3′-UTR sequences of isolated EV-G strains were compared to genome organization of prototype EV-G1 and recombinant EV-G strains. The VP1 of isolated EV-G strains consists of 729 nt and encodes a protein of 243 aa. The VP1 gene of isolated EV-Gs shared sequence similarities with those of prototype EV-G1 strains ranging from 72.7% to 79.8% nt and from 83.5% to 85.2%% aa with CH/17GXQZ/2017 and UKG/410/73, respectively. Compared with other chimeric EV-G1, EV-G2, and EV-G17 strains, the isolated EV-G strains showed relatively high VP1 sequence homology with chimeric EV-G1 strains ranging from 80.4% to 90.3% nt and from 93.4% to 96.7% aa, but exhibited lower 61.2%–61.3% nt and 59.7%–64.6% aa identities with Japanese EV-G2 and US EV-G17 strains, respectively (Table 3). Furthermore, the genome sequences of isolated EV-G strains shared 98.8%–100.0% nt and 99.5%–100.0% aa sequence similarities with one another (Table S2).

**TABLE 3:** Comparison of full-length genomes of the isolated strains with EV-G reference strains

Genome regions of isolated strains	nt and aa similarities of different strains								
	MF782664 (PLCP)	MH663501 (PLCP)	KY214435 (PLCP)	LC316778 (PLCP)	KY498016 (PLpro)	MT274669 (G1)	Y14459 (G1)	LC316791 (G2 PLCP)	KY761948 (G17 PLCP)
5′-UTR (813 nt)		97.5 (−)	92.5 (−)	94.1 (−)	85.9 (−)	87.8 (−)	82.2 (−)	93.2 (−)	94.3 (−)
VP4 (207 nt)		90.3 (94.2)	78.3 (91.3)	89.9 (95.7)	72.9 (78.3)	76.3 (87.0)	85.0 (88.7)	71.0 (81.2)	78.3 (91.3)
VP2 (738 nt)		86.9 (96.3)	79.4 (92.3)	86.4 (93.1)	83.1 (95.1)	73.6 (90.7)	80.6 (93.9)	71.3 (83.7)	70.3 (83.7)
VP3 (831 nt)		90.4 (96.8)	81.7 (94.6)	88.6 (97.5)	87.8 (95.4)	75.2 (89.4)	81.5 (91.7)	67.1 (70.4)	67.3 (71.8)
VP1 (729 nt)	90.3 (96.7)	87.1 (95.5)	80.4 (93.4)	88.1 (94.7)	85.5 (95.5)	72.7 (83.5)	79.8 (85.2)	61.2 (59.7)	61.3 (64.6)
2A (450 nt)		83.1 (93.3)	82.9 (92.7)	83.3 (93.3)	83.8 (96.7)	82.4 (94.7)	82.6 (79.3)	83.6 (95.3)	84.7 (94.7)
2B (297 nt)		88.6 (98.0)	86.5 (98.0)	88.2 (99.0)	83.5 (97.0)	70.7 (81.8)	76.1 (82.8)	87.5 (100.0)	88.6 (96.0)
2C (987 nt)		89.8 (99.7)	88.3 (97.6)	91.2 (99.1)	86.3 (97.9)	78.7 (90.6)	76.5 (86.6)	87.8 (99.4)	86.3 (97)
PLCP (639 nt)	651nt (84.1) (91.1)	594 (79.6) (89.8)	636 (83.2) (83.4)	645 (87.5) (92.5)	669 (81.5) (84.5)	/	/	633 (85.7) (87.2)	582 (78.6) (89.6)
3A (267 nt)		90.3 (96.6)	85.4 (96.6)	90.3 (98.9)	87.6 (95.5)	72.7 (87.6)	76.0 (88.8)	85.0 (100.0)	83.9 (97.8)
3B (66 nt)		80.3 (100.0)	83.3 (100.0)	72.7 (100.0)	81.8 (100.0)	80.3 (95.5)	78.8 (95.5)	84.8 (100.0)	80.3 (100.0)
3C (549 nt)		88.9 (99.5)	87.1 (97.8)	88.9 (98.9)	86.7 (99.5)	72.7 (83.6)	71.9 (84.2)	86.5 (99.5)	84.3 (97.3)
3D (1383 nt)		91.7 (99.1)	84.8 (98.3)	92.4 (98.5)	89.4 (98.5)	78.4 (87.6)	79.8 (88.5)	86.3 (97.0)	86.3 (97.2)
3′-UTR (71 nt)		97.2 (−)	98.6 (−)	98.1 (−)	92.6 (−)	88.4 (−)	87.0 (−)	96.3 (−)	98.6 (−)
Complete genome with PLCP	92.2	90.7	86.0	90.4	86.3	78.1	79.7	81.6	81.7

### Phylogenetic analysis of isolated EV-G strains

**FIG 3:**
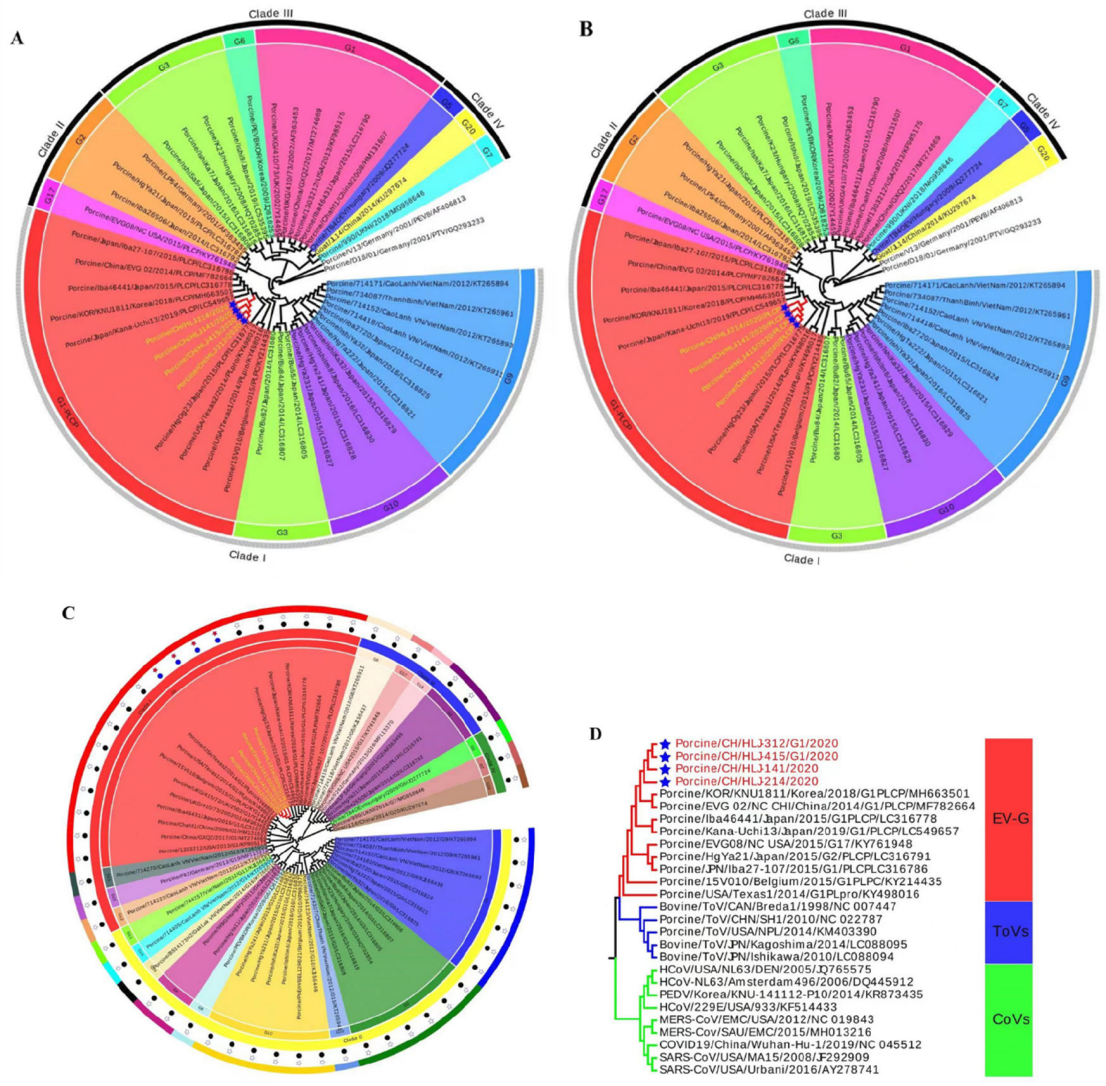
Phylogenetic analyses based on nucleotide sequences of the complete genome (**A**), complete genome excluding insertion PLCP sequences of EV-G strains (**B**), VP1 gene sequences (**C**), and phylogenetic analyses based on *PLCP* genes of EV‐G strains and nidoviruses including toroviruses (ToVs) and coronaviruses (CoVs) (**D**). Multiple sequence alignments were created using the ClustalX v.2.0 program, and the phylogenetic trees were constructed from the aligned nucleotide sequences using neighbor‐joining. Hosts of origin, geographical origins, names of the strains, years of isolation, genotypes, and GenBank accession numbers are shown. The genotypes are indicated on the right‐hand side. Solid diamonds denote the recombinant EV‐G1‐PLCP strains identified in this study. Scale bars indicate nucleotide substitutions per site.

The phylogenetic tree based on entire genome sequences was constructed using sequences of the isolated EV-G strains and representative EV-G strains from the NCBI database (Table S3). The results showed that EV-G strains were phylogenetically classified into four clades (clades I, II, III, and IV), and EV-G sequences retrieved in the current study were grouped together with EV-G1-PLCP strains (Fig. 3A). Moreover, EV-G strains isolated herein have a common ancestor within clade I that included EV-G3, EV-G9, and EV-G10 genotypes according to the results, but are phylogenetically distinct from prototypic G1 strains, which were classified under clade III. Interestingly, phylogenetic analysis of the entire genome, omitting the PLCP sequence from all recombinant strains, showed the same topological structure (Fig. 3B). These findings indicate that the insertion is the result of viral recombination, and substantial nucleotide differences between related EV-G genotypes have arisen throughout the whole genome.

The molecular classification of EV genotypes is solely based on capsid-encoding sequence (VP1) (42, 43). Therefore, phylogenetic analysis based on EV-G VP1 sequence of isolated EV-G strains and reference sequences was carried out. The EV-G strains isolated during this study were grouped with the genotype (G1) and closely related with chimeric EV‐G1 strains (Fig. 3C). Furthermore, phylogenetic analysis of the *PLCP* genes of EV-Gs and coronaviruses revealed that the *PLCP* gene of EV-G isolated in this study formed a cluster with PLCPs of other EV-Gs (Fig. 3D) and more closely related to the ToVs, but were distantly related to strain of swine, bovine, and equine nidoviruses, showing lower sequence similarities (46%–58.7%) in the aa sequence.

### Cell susceptibility test of the EV-G isolate

**FIG 4:**
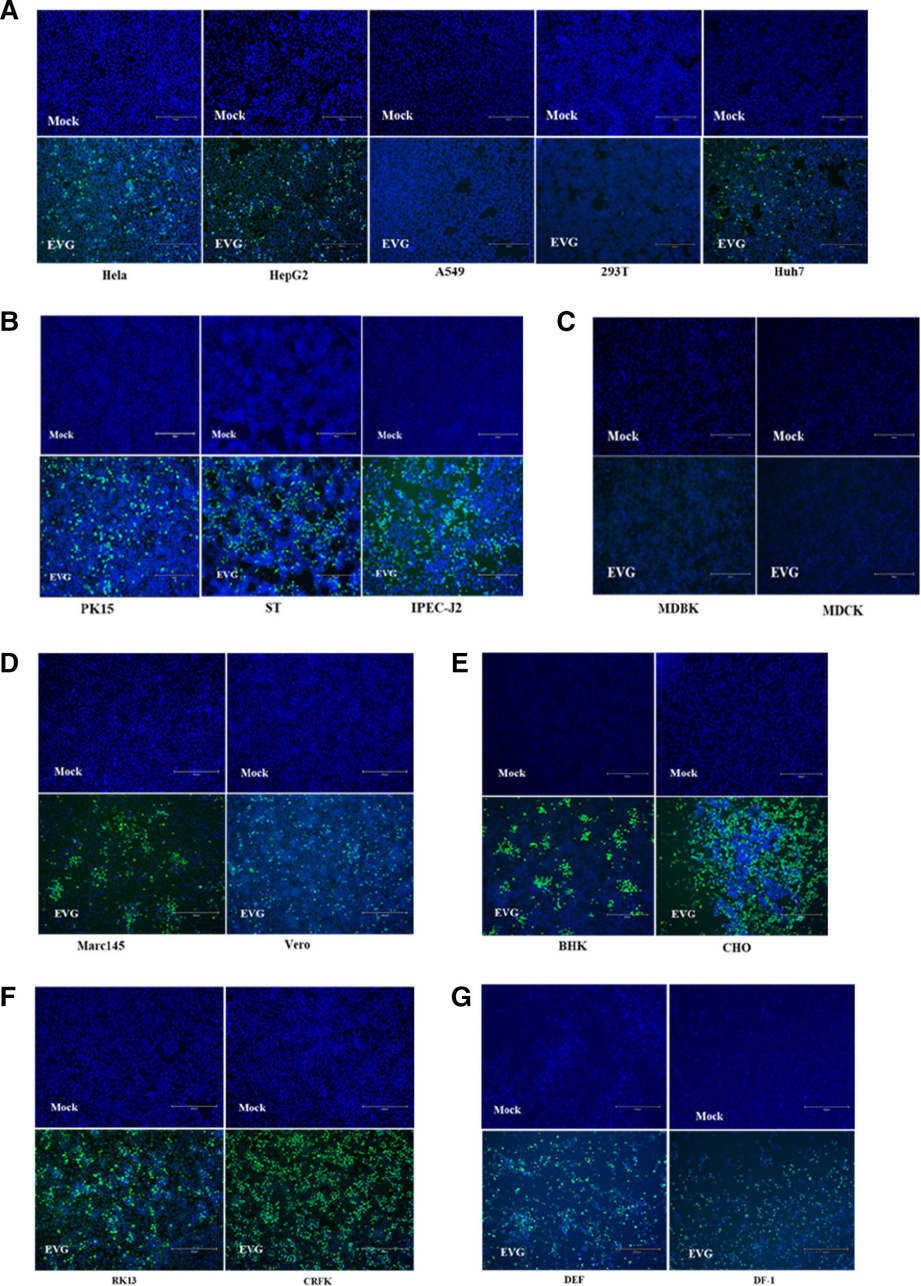
Susceptibility of EV-G to cell lines. Immunofluorescence assay of cells infected with EV-G at an MOI of 0.01 at 12 hpi was performed using mouse anti-EV-G polyclonal antibody as primary antibody and fluorescein isothiocyanate (FITC)-conjugated goat anti-mouse IgG as secondary antibody, with 4′,6-diamidino-2-phenylindole (DAPI) for visualization of cell nuclei. Mock-infected cells were treated with the same procedures as appropriate. Cells lines derived from various species including (**A**) human (HeLa, HepG2, A549, 293T, and Huh-7); (**B**) swine (PK15, ST, and IPEC-J2); (**C**) bovine (MDBK) and canine (MDCK); (**D**) monkey (Marc-145 and Vero); (**E**) hamster (CHO and BHK-21); (**F**) rabbit (RK13) and feline (CRFK); and (**G**) duck (DEF) and chicken (DF-1) were tested.

To examine the host tropism of the isolated EV-G strains, 18 cell lines derived from various species were tested with the CH/HLJ-214/G1PLCP/2020 strain. The result revealed that 14 out of 18 cell lines, namely, porcine (PK15, ST, and IPEC-J2), human (Huh7, HepG2, and Hela), monkey (Marc145 and Vero E6), feline (CRFK), rabbit (RK13), hamster (BHK21 and CHO), duck (DEF), and chicken (DF-1) cell lines showed CPEs at 24-h post-infection (hpi), whereas the human (293T and A549), bovine (MDBK), and canine (MDCK) cell lines did not show CPEs (Table 4). Evidence of infection ability of the isolated EV-G strain in the susceptible cell lines was confirmed by the detection of viral VP1 protein expression by using immunofluorescence assay (IFA). As shown in Fig. 4, the VP1 protein was expressed in all tested cell lines except the human (293T and A549), bovine (MDBK), and canine (MDCK) cell lines, which was consistent with the CPE results.

**TABLE 4:** Summary of cell lines and their susceptibility to porcine sapelovirus infection as determined by CPE and IFA^*a*^

Cells						
No.	Name	ATCC no.	Tissue origin	Species	CPE	IFA
1	293T	CRL-11268	Embryonic kidney	Human	−	−
2	A549	CCL-185EMT	Lung carcinoma	Human	−	−
3	Hela	CCL-2	Cervix adenocarcinoma	Human	+	+
4	HepG2	HB-8065	Hepatocellular carcinoma	Human	+	+
5	Huh7	N/A	Hepatocellular carcinoma	Human	+	+
6	PK15	CCL-33	Porcine Kidney	Swine	+	+
7	ST	CRL-1746	Swine testicular	Swine	+	+
8	IPEC-J2	N/A	Small intestinal epithelium	Swine	+	+
9	Marc145	N/A	African green monkey kidney	Monkey	+	+
10	Vero E6	CRL-1586	African green monkey kidney	Monkey	+	+
11	MDBK	CCL-22	Madin-Darby bovine kidney	Bovine	−	−
12	MDCK	CCL-34	Madin-Darby canine kidney	Canine	−	−
13	CRFK	CCL-94	Crandell-Rees feline kidney	Feline	+	+
14	RK13	CCL-37	Rabbit kidney	Rabbit	+	+
15	BHK-21	CCL-10	Baby hamster kidney	Hamster	+	+
16	CHO	CCL-61	Chinese hamster ovary	Hamster	+	+
17	DEF	CCL-141	Duck embryo fibroblasts	Duck	+	+
18	DF-1	CRL-12203	Embryo fibroblasts	Chicken	+	+

^*a*^ N/A, not available; +, infection; −, no infection or obvious lesion.

### Pathogenicity evaluation of the EV-G isolate

**FIG 5:**
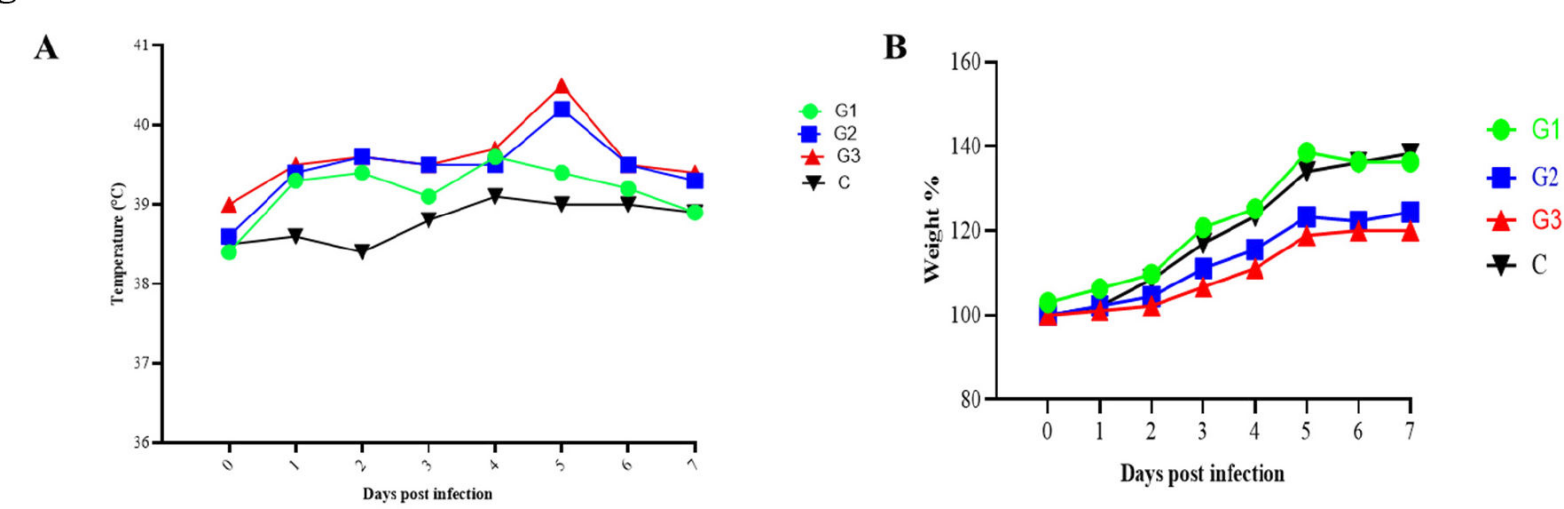
Animal experiments. (**A**) Trends of rectal temperatures of four 21-day-old piglets inoculated with isolated EV-G. Normal rectal temperature was present in the mock (**C**), while the experimentally inoculated pigs (G1, G2, and G3) exhibited pyrexia (39.5–40.2°C) at 2 days post-inoculation, which persisted for 2–6 days. (**B**) Trends weight gain of piglets inoculated with EV-G. The weight gain rates in piglets from the infected group (G2 and G3) were less than those in control piglet (**C**).

**FIG 6:**
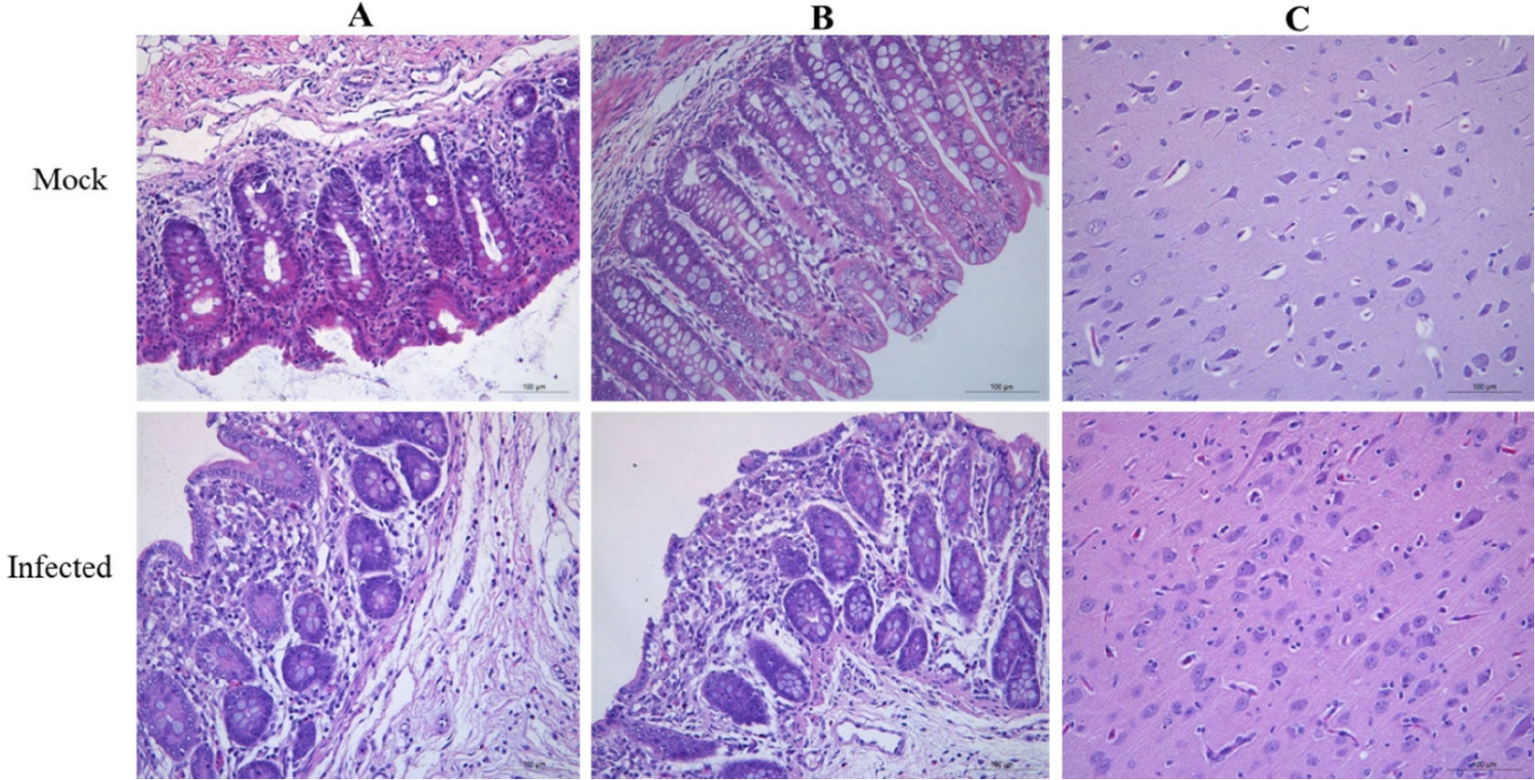
Histopathologic changes observed in the cecum, colon, and cerebrum of piglets infected with EV-G in mock and the infected groups. (**A**) The cecum showing submucosa edema. (**B**) Colon-lamina propria and submucosa showing edema, a small amount of degeneration and necrosis of mucosal epithelial cells. (**C**) The microscopic lesions observed in the cerebrum of piglets show glial cell hyperplasia; neutrophilic phenomenon can be seen, as well as some neuronal cell degeneration and necrosis.

**FIG 7:**
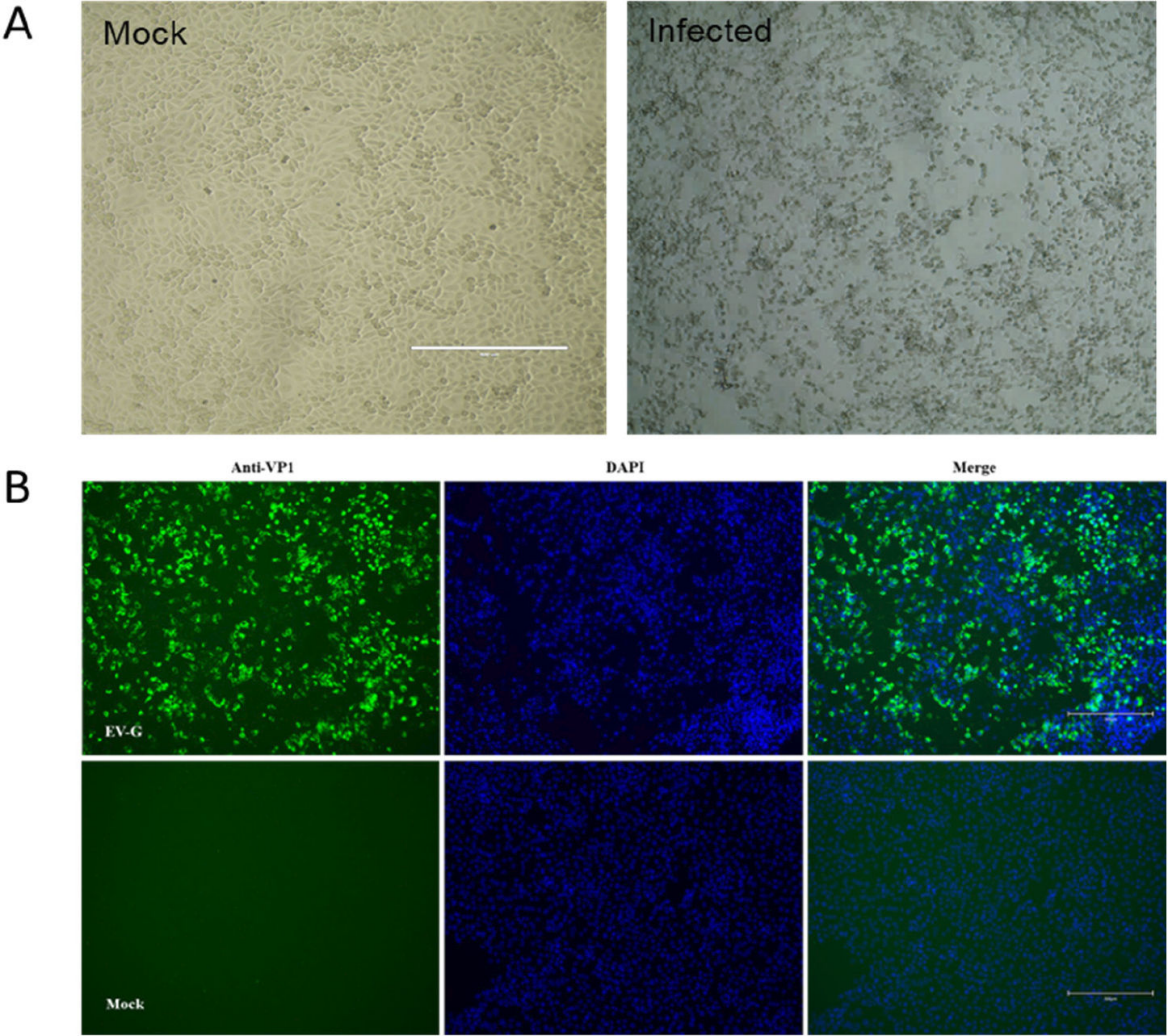
EV-G isolation from infected piglets. (**A**) Virus re-isolation on Marc145 cells inoculated with suspensions of tissues from infected piglets (e.g., cecum and colon) shown with CPE. (**B**) Immunofluorescence assay of Marc145 cells infected with re-isolated EV-G at 12 hpi, using mouse anti-EV-G polyclonal antibody as primary antibody and FITC conjugated goat anti-mouse IgG as secondary antibody, with DAPI for visualization of cell nuclei. Mock-infected cells were treated with the same procedures as appropriate.

To determine whether the isolated EV-G is pathogenic in piglets, three 2-week-old specific-pathogen-free (SPF) piglets were inoculated with CH/HLJ-214/G1PLCP/2020 strain. After virus inoculation, all three EV-G-inoculated piglets showed temporary diarrhea. Two piglets exhibited pyrexia (39.5–40.5°C) at 2 days post-inoculation (dpi), which persisted for 2–7 days (Fig. 5A). Similarly, the same two piglets gained less weight at 4–7 dpi compared to the control piglet (Fig. 5B). Daily fecal swabs and tissue samples from all piglets were collected to investigate virus shedding and distribution using RT-PCR. The results demonstrated that EV-G was detected in fecal samples of all three infected piglets at 2–7 dpi but not in the control piglet (Fig. S2a). Furthermore, viral RNAs were detected in most tested tissues including blood, spinal fluid, tonsil, pancreas, ileum, colon, rectum, cerebrum, and cerebellum, but not in lung, liver, and kidney from the infected pigs (Fig. S2a and b). The necropsy results showed that the wall of small intestine of EV-G-infected piglets became thinner and translucent; the intestinal contents were mushy or watery; and there was obvious congestion compared to the control piglet (Fig. S3a); mild hyperemia was observed in the cerebrum of infected piglets (Fig. S3b). Tissues of infected animals were also histopathologically examined, and results showed edema, a small amount of degeneration, and necrosis of mucosal epithelial cells in the lamina propria and submucosa of the cecum and colon. Similarly, glial cell hyperplasia, neutrophil infiltration, and some neuronal cell degeneration and necrosis, which led to an increase in nearby glial cells, were observed in the cerebrum of infected piglets; however, there was no significant change in the control group (Fig. 6). Furthermore, the virus re-isolation from EV-G-infected intestinal contents showed obvious CPE on Marc145 cells at 24 hpi, which was confirmed by IFA with the specific antibody against VP1 of EV-G as shown in Fig. 7A and B.

### Epidemiological investigation of EV-Gs

To assess the frequency of EV-G infection among swine, a total of 232 porcine fecal samples, collected from 134 diarrheic and 98 non-diarrheic pigs, were examined by RT-PCR. As shown in Table 5, out of 232 samples, 37.5% (87 of 232) samples were positive for EV-G; of these EV-G-positive pigs, 44% (59 of 134) were diarrheic and 28.6% (28 of 98) were non-diarrheic. Furthermore, EV-G was identified in 21.4% (19 of 89) of the suckling pigs, 52.9% (45 of 85) of the nursery pigs, and 39.7% (23 of 58) of the fattening pigs. The EV-G-positive rates in the nursery and fattening groups were significantly higher than those in the suckling piglets. The data suggest that the prevalent rate of EV-G was clearly higher in diarrheic animals than in non-diarrheic animals.

**TABLE 5:** Prevalence of EV-G infection in diarrheic and asymptomatic animals

	Clinical status		Total (***N*** = 232)
Production stage	Diarrheic (***n*** = 134) ***n*** (%)	Asymptomatic (***n*** = 98) *n* (%)	*n* (%)
Suckling			
Positive	13 (24.1.0)	6 (17.1)	19 (21.4)
Negative	41 (75.9)	29 (82.9)	70 (78.7)
Nursery			
Positive	29 (61.7)	16 (42.1)	45 (52.9)
Negative	18 (38.3)	22 (57.9)	40 (47.1)
Fattening			
Positive	14 (42.4)	9 (36.0)	23 (39.7)
Negative	19 (57.6)	16 (64)	35 (60.3)
Total			
Positive	59 (44.0)	28 (28.6)	87 (37.5)
Negative	75 (56.0)	70 (71.4)	145 (62.5)

## DISCUSSION

Recently, the identification and characterization of a natural recombination EV-Gs with insertion of the *PLCP* gene in their genomes from stool samples of swine with diarrhea have been increasingly reported (26, 38 – 41, 44), indicating that EV-G-PLCP might be the potential causative agent of porcine diarrhea. The viral evolution and recombination events may have a significant impact on a variety of factors, including the emergence of novel virus variants, changes in host ranges and tissue tropism alterations, increases in virulence, and evasion of host immunity (45 – 47). A previous study has reported that ToV-PLCP might have an impact on EV-G pathogenesis by functioning as an innate immune response antagonist (39). Thus, the finding of *PLCP* gene insertions in EV-G may increase the potential public health risks. In this report, four recombinant EV-G strains, named CH/HLJ-141/G1PLCP/2020, CH/HLJ-214/G1PLCP/2020, CH/HLJ-312/G1PLCP/2020, and CH/HLJ-315/G1PLCP/2020, were isolated from diarrheic piglets. The genetic and biological characteristics of isolated viruses were determined by using several techniques including RT-PCR, sequencing, CPE, IFA, replication kinetics, and TEM. Furthermore, the pathogenicity of isolated EV-G strains in piglets was evaluated.

Since four isolated viruses have relatively similar characteristics, further experiments were carried out with the representative strain CH/HLJ-214/G1PLCP/2020. The epidemiological data on EV-G infections are limited; thus, this study also provided novel information about the prevalence of EV-G in diarrheic and non-diarrheic swine. Most studies reported that EV-Gs cause asymptomatic infections in swine; for example, in Germany, Hungary, Japan, and Vietnam, EV-G3, EV-G4, EV-G8, EV-G9, and EV-G10 were detected in healthy pigs (21, 23, 26, 44, 48). In contrast, our result revealed that the overall prevalence of EV-G was much higher in diarrheic than in non-diarrheic animals. In addition, the highest prevalence was found in nursery pigs followed by fattening pigs, which was consistent with a study from Thailand (27). Moreover, several studies have reported that EV-G is prevalent in swine populations, and infection is detected at higher frequencies in younger pigs compared to adults, which might be attributed to acquired immunity (23, 24, 49, 50).

The phylogenetic analysis based on structural protein sequences is widely used to determine picornavirus taxonomy (51, 52). To understand the molecular characteristics of the isolated EV-G strains, complete genomes were sequenced and phylogenetic relationships among representative EV-G strains were determined. The sequence analyses reveal that all four isolated EV-Gs contain a *PLCP* gene of 639 nt within the 2C/3A junction region of their genome and encode a protein similar to torovirus PLCP, which resembles the picornavirus leader protease. Consistent with our study, recombinant EV-G strains (EV-G1, EV-G2, and EV-G17) carrying PLCP in the junction region 2C/3A of their genomes have been detected in pigs with diarrhea in the USA, Belgium, Japan, South Korea, and China (26, 38 – 41, 44, 53). Interestingly, Wang et al. have identified one recombinant EV-G strain carrying the torovirus *PLCP* gene, completely replacing the viral capsid protein gene region (VP4/VP2/VP3/VP1) in pigs in China (41). The previous study has reported that the 3C protease of EV-G cleaves the viral polyprotein precursor at the C-terminus of 2C protein and N-terminus of 3A proteins by using the cleavage sequence ALFQ↓GPPT (54). Moreover, Shang et al. have demonstrated that the chimeric EVG 08/NC_USA/2015 expressing PLCP using the reverse genetic technique can produce the exogenous PLCP protein at the cleavage sites of ALFQ↓GPPV and AEFQ↓GPPT in the virus-infected cells (39). According to the sequence similarity of the recombinant EV-G strains, the possible cleavage residues flanking PLCP includes GPPT↓ALFQ, GPPA↓ALFQ, and GPPE↓ALPQ (26, 38, 44). In this study, the PLCP in the isolated recombinant EV-G strains is flanked by the predicted viral 3C protease cleavage sequences, ALFQ↓GPPA and AVFQ↓GPPT, at its N-terminus and C-terminus, respectively, indicating that the viral 3C protease of the recombinant EV-G isolates can process the functional PLCP protein.

The phylogenetic analysis based on complete genome revealed that the EV-G strains isolated in this study grouped together with global G1-PLCP strains and more closely related to EV-G/PLCP strains previously detected in China, Japan, and Korea with (90.3%–92.2% nucleotide similarities) than other recombinant EV-Gs, implying that the isolated EV-Gs here may have originated from the common ancestor. However, all the recombinant G1-PLCP strains, including four strains isolated here, were not phylogenetically close to the G1 strains but were grouped together with the large clade of enteroviruses containing EV-G genotypes G3, G9, and G10. Additionally, phylogenetic analysis of all recombinant strains excluding the *PLCP* gene resulted in the same evolutionary tree. These data suggested that the PLCP insertion has occurred through cross-order recombination, and the nucleotide variations of EV-G entire genome have arisen from the accumulation of single-base changes or the recombination of different genotypes during viral propagation. Phylogenetic analysis of *PLCP* genes showed that the *PLCP* gene of EV-G isolated here are more closely related to that of the Japan/LC316778 strain (92.5% aa similarity), establishing a cluster with PLCPs from other EV-Gs. Despite the pathogenicity of EV-Gs in swine remaining debatable, all the isolated EV-G/PLCP strains here have been identified from fecal samples of swine with diarrhea, indicating that EV-G-PLCP is the potential causative agent of diarrhea in swine.

PLCP was previously shown to decrease the host cells’ innate immune response when introduced into the EV-G genome (39), which, under specific conditions, may allow them to demonstrate their pathogenic potential. Furthermore, it has been noted that recombinant events in the EV-G2 and EV-G17 genotypes are infrequent, but they appear to occur more frequently in the EV-G1 genotype, which may play a key role in virus evolution (40). Given the fact that human EVs are frequently discovered in several mammalian species, it is plausible that EVs which naturally circulate in animal populations may potentially infect human populations as well. Due to strain diversity, a high rate of mutation, prolonged subclinical shedding, low infectious doses, and frequent genome recombination of enterovirus (55), there is a significant potential for the emergence of new strains that can infect and replicate in a wide range of hosts. To evaluate the potential cross-species infection ability of EV-G, 18 cell lines derived from various host species, including human, swine, monkey, hamster, bovine, dog, cat, rabbit, chicken, and duck, were subjected to a susceptibility study. Previous reports showed that EV-G can be propagated in BHK-21, Vero, ST, and Marc- 145 cells in the presence of trypsin (13, 56). Our results demonstrated that EV-G could infect and replicate in a wide range of cell lines including swine (PK15, ST, and IPEC-J2), human (Huh7, HepG2, and Hela), monkey (Marc145 and Vero E6), feline (CRFK), rabbit (RK13), hamster (BHK21 and CHO), duck (DEF), and chicken (DF-1). These various levels of susceptibility should help define the EV-G receptors. Additionally, the wide range of cells that the isolated EV-G strains can infect *in vitro* suggests that EV-Gs may pose a risk of cross-species infection.

Although the EV-Gs are frequently isolated from healthy pigs and have not been proved to cause disease, some reports indicate that EV-G infection in infected animals may induce clinical symptoms such as dermatitis, reproductive problems, neurological problems, and diarrhea (23, 57, 58). However, there is minimal evidence that EV-G infection causes clinical diarrhea. To date, the pathogenicity of EV-G has been poorly studied, especially the recombinant EV-G strains, and only two EV-G trains have been subjected to pathogenicity assessment (24, 34). Since CH/HLJ-214/G1PLCP/2020 can infect broad types of cell lines from different species *in vitro* and the predicted precursor polyprotein of CH/HLJ-214/G1PLCP/2020 is relatively close to other EV-G isolates in China, therefore, further animal experiments were carried out with the representative strain CH/HLJ-214/G1PLCP/2020. To determine the pathogenicity of the recombinant EV-G strain isolated in the present study, four SPF piglets were infected. The results revealed that two piglets showed mild diarrhea, pyrexia, and lower body mass index. Additionally, most tissues of the infected piglets carried EV-G, which could be detected at 7 dpi. Even though no piglets died during the entire experimental infection process, it is important to note that the EV-G strains isolated herein could negatively impact the growth rate of piglets by reducing weight gain. The results of tissue histological examination revealed that isolated EV-G could cause pathological changes in the cecum, colon, and cerebrum, which is similar to the animal experiment by using EV-G stains without the *PLCP* gene (24, 34). These findings support that the EV-G isolate carrying ToV *PLCP* gene is pathogenic, which might be one of the causative agents for swine diarrhea.

In summary, this research will provide trustworthy data on the biological properties, evolution, and pathogenicity of recombinant EV-G strain isolated from diarrheic piglets. The fact that isolated EV-Gs can infect cell lines from different species suggests that they may pose a risk of cross-species infection. Therefore, it is important to recognize that EV-Gs may have the potential to infect multiple species, and further research is needed to understand this risk.

## MATERIALS AND METHODS

### Sample collection

In July 2020, 15 fecal samples of piglets with diarrhea were collected from swine farm in northeast of China. In addition, a total of 232 fecal samples (with or without clinical diarrhea) were collected from different swine age groups (suckling, nursery, and fattening) in northeast of China (Table 6). All samples were stored at −80°C until use.

**TABLE 6:** Fecal sample background of EV-G

Production stage	Clinical status		Total
	Diarrheic	Asymptomatic	
Suckling (<28 days)	54	35	89
Nursery (28–70 days)	47	38	85
Fattening (>70 days)	33	25	58
Total	134	98	232

### Cell cultures

Eighteen cell lines derived from different species including pig, human, monkey, bovine, canine, feline, rabbit, hamster, duck, and chicken were used (see Table 4). Marc145 cell was cultivated in RPMI-1640 medium (Gibco); DEF and DF-1 cells were cultured in Eagle's minimum essential medium (EMEM; Multicell), and the remaining cells were cultured in Dulbecco's modified Eagle medium (DMEM; Gibco). All media were supplemented with 10% fetal bovine serum (FBS; Biological Industries) and penicillin (100 U/mL)-streptomycin (100 µg/mL).

### Isolation of EV-G

To identify the causative agents of swine diarrhea, fecal samples were diluted in phosphate-buffered saline (PBS) to prepare a 10% fecal suspension. The fecal suspensions were clarified by centrifugation and filtered through a 0.22-µm filter (Merck Millipore, Burlington, MA, USA). The samples initially were examined for common swine enteropathogenic viruses, including TGEV, PEDV, PDCoV, and rotavirus genogroup A using RT-PCR. Five out of 15 samples were tested negative for these enteric viral pathogens. The negative samples were further tested for porcine astrovirus (AstV), porcine sapovirus (PSaV), porcine kobuvirus (PKV), porcine sapelovirus (PSV), and EV-G using virus‐specific primers as previously described (59 – 62). Only four samples were tested positive for EV-G alone. These four samples were then subjected to virus isolation. In brief, 1 mL of filtered fecal supernatants from each sample was pre-treated with trypsin (Gibco) at a final concentration of 20 µg/mL for 1 h, diluted at 1:1 ratio with complete DMEM (containing 1% penicillin-streptomycin), and then inoculated onto confluent Marc145 cells for 1 h. The inoculum was discarded; cell monolayers were washed twice with PBS and covered with fresh DMEM supplemented with 5 µg/mL trypsin; and observed daily for CPEs. Four days post-infection, cells were lysed by freeze-thawing three times and re-inoculated into respective Marc145 cells for three passages; at the same time, the growth of the EV-G in culture was further confirmed by RT-PCR with specific primers after every passage.

### Viral plaque assay

To purify the isolated viruses, a plaque assay was performed as previously described with slight modifications (63). In brief, Marc145 cells were inoculated with serially diluted viruses and overlayed with agarose medium. After the overlaid medium was solidified, the plates were incubated at 37°C and 5% CO_2_. Plaques were allowed to develop for 5–7 days, and uniform plaques were picked and re-inoculated into cell monolayers to amplify the positive clones. After four rounds of plaque purification, the purified virus clones were successfully obtained. The monolayers were also fixed and stained with crystal violet to visualize the viral plaques.

### TEM

The transmission electron microscopy was carried out according to the previous publication (63). The cell supernatants of plaque-purified viruses were harvested after three times freezing-thawing, and cell debris was clarified by centrifuging at 10,000 rpm for 30 min at 4°C. The supernatants were passed through a 0.22-µm filter and pelleted by centrifuging at 35,000 rpm for 4 h (Beckman SW32Ti rotor). The resulting pellet was re-suspended in DMEM and centrifuged through 20%–50% sucrose cushion at 35,000 rpm for 4 h. The white opalescent band at the interface formed by the virus particles were collected and re-centrifuged at 35,000 rpm for 3 h to get rid of sucrose and concentrate samples. The purified virus was examined under TEM (Hitachi Model H-7650).

### Virus growth kinetic

To analyze the growth properties of isolated virus, the growth kinetics of isolated virus was evaluated in the same cell line used for virus isolation to avoid influences of adaptive mutations acquired through the virus isolation. In brief, Marc145 cells were cultured in 12-well plates to 90%–100% confluence; cells were washed twice by PBS after discarding the growth medium; the cells were infected in triplicate with EV-G at an MOI of 0.01 and incubated at 37°C and 5% CO_2_ for 1 h. After the incubation period, the inoculum was discarded and cells were washed as above, then 1 mL of maintenance media was added and cells were incubated at 37°C and 5% CO_2_. Cell supernatants were harvested at 1, 6, 12, 24, 36, 48, 60, and 72 h post-inoculation and stored at −80°C until use. Virus titers were measured in Marc145 cells by TCID_50_ according to the Reed-Muench method (64).

### Whole-genome sequencing and analysis

The EV-G supernatants obtained from plaque-purified clones were clarified by centrifuging at 10,000 rpm for 30 min to remove cell debris. Total RNA was extracted using the TIANamp RNA Kit and cDNA was made using the PrimeScript Synthesis Kit (Takara, Dalian, China). The cDNA libraries were prepared from total RNA extracted from each isolate using the Agencourt AMPure XP-Medium kit (A63881, Beckman Coulter, USA) according to the manufacturer’s instructions. The product was validated on the Agilent Technologies 2100 Bioanalyzer for quality control. The final qualified libraries were subjected to next-generation sequencing using BGISEQ-500 Sequencing System (BGI-Shenzhen, China). Based on the reference genome of *Sus scrofa* (Sscrofa11.1(GCA_000003025.6)), the high-quality non-host reads that matched to the candidate viruses were processed by eliminating host contamination reads using BWA and SOAP (65). The high-quality reads were then *de novo* assembled by using IDBA (66), SPAdes (67), and Edena (68) software. The assembled contigs were then analyzed by BLAST-based approach to identify the virus species (69).

To further understand the molecular characteristics of isolated EV-G strains, the complete ORF and cleavage sites of isolated EV-G were predicted by comparing with the genome of previously known EV-G strains. Furthermore, the nucleotide and aa sequences of the whole genome, *VP1*, and *PLCP* genes were aligned with reference EV-G strains using Clustal W software (70). MEGA v.6.0 software was used to analyze the phylogenetic trees of whole-genome, VP1, and PLCP sequences via the maximum-likelihood method with the neighbor-joining method with Kimura 2-parameter and 1,000 bootstrap replicates (71, 72).

### Cell susceptibility test to the EV-G

To determine the viral tropism, 18 cell lines derived from different species were subjected to the isolated EV-G strain and observed for CPEs. An IFA was then performed to determine the production of viral VP1 protein as described previously (63). Briefly, 18 cell monolayers were inoculated with the EV-G for 12 h and then fixed with paraformaldehyde. The fixed cells were stained with mouse polyclonal antibody specific for EV-G-VP1 protein and FITC-conjugated goat anti-mouse IgG (Thermo Fisher Scientific). The cell monolayers were then stained with DAPI (Solarbio, China) and visualized under a digital inverted fluorescence microscope (Evos, FL, USA).

### Animal experiments

To evaluate the pathogenicity of the isolated EV-G, four 2-week-old SPF piglets, negative for EV-G, ASFV, PRRSV, PCV2, PSV, PEDV, PDCoV, TGEV, rotavirus, PSaV, PKV, AstV and mammalian orthoreovirus in the fecal samples by RT-PCR, were housed in a HEPA-filtered level 2 biosecurity facility and divided into two groups. Piglets in group 1 (named G1, G2, and G3) were challenged orally with 5-mL 5 × 10^6^ TCID_50_/mL of CH/HLJ-141/2020 /G1/PLCP strain each, while piglets in group 2 (named C1) received 5-mL DMEM orally as a negative control. The piglets were monitored daily, and rectal temperature, clinical signs, and weight gain were recorded. In addition, fecal swabs and blood samples were collected at 0–7 dpi, and serum samples were collected for seroconversion tests at 1, 3, and 6 dpi. All piglets were euthanized at 7 dpi, and the tissues from the heart, liver, spleen, lung, kidney, tonsil, cerebellum, cerebrum, submaxillary nodes, inguinal lymph nodes, and alimentary tract, including the stomach, duodenum, jejunum, ileum, caecum, colon, rectum, and mesenteric lymph nodes as well as fecal, blood, and spinal fluids were collected. All samples taken from inoculated piglets were exposed to viral RNA detection to determine the virus shedding and distribution in the infected piglets. Additionally, certain tissue samples were immediately preserved in 10% neutral buffered formalin for histological investigation.

### RT-PCR detection of EV-G

To assess the frequency of EV-G infection in pigs, a molecular survey was conducted on 232 stool samples, including feces and fecal swabs collected from 98 non-diarrheic and 134 diarrheic pigs. Fecal samples were diluted in PBS to prepare a suspension of 10% (wt/vol). Rectal swabs were processed by elution into 0.5-mL PBS and centrifuged at 14,000 × *g* for 10 min. The total viral RNA was extracted from supernatants using RNA extraction kit (Tiangen Biotech, Beijing) according to the manufacturer’s instructions. The cDNA synthesis was carried out in a 20-µL reaction mixture using murine leukemia virus (MLV) reverse transcriptase kit (Takara), following the manufacturer’s instructions. PCR was used to detect EV-G genome using particular primers based on a conserved sequence within the 5′-UTR as previously reported (61).

## Data Availability

The enterovirus G strains obtained in this study are available in GenBank under accession numbers ON964506 for CH/HLJ-141/G1PLCP/2020, ON964507 for CH/HLJ-214/G1PLCP/2020, ON964508 for CH/HLJ-312/G1PLCP//2020, and ON964509 for CH/HLJ-315/G1PLCP//2020.

## SUPPLEMENTAL MATERIAL

Supplemental material spectrum.02643-23-s0001.docx Fig. S1 to S3; Tables S1 to S3.

ASM does not own the copyrights to Supplemental Material that may be linked to, or accessed through, an article. The authors have granted ASM a non-exclusive, world-wide license to publish the Supplemental Material files. Please contact the corresponding author directly for reuse.
