# Supplementary material for: Molecular characterization and pathogenicity evaluation of enterovirus G isolated from diarrheic piglets
Source: Microbiol Spectr. 2023 Oct 13;11(6):e02643-23. doi: 10.1128/spectrum.02643-23 (PMC10715025; doi:10.1128/spectrum.02643-23)
Supplement: Supplemental material — Fig. S1 to S3; Tables S1 to S3. [file spectrum.02643-23-s0001.docx]

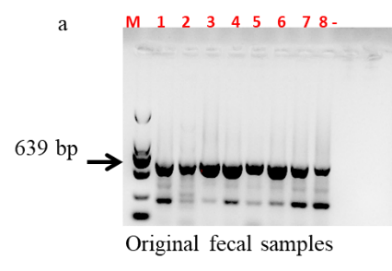

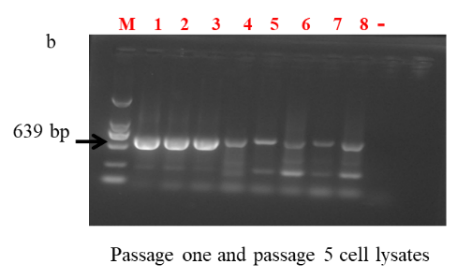

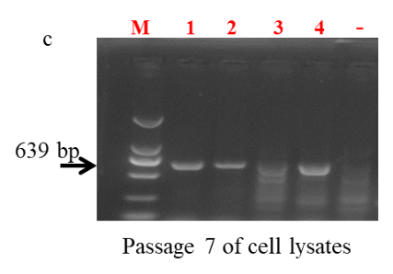


**Fig. S1** Amplification of PLCP insertion (639 bp) by RT-PCR. (a) original fecal samples; (b) passage one and five of Mar145 cells infected with isolated EV-Gs; (c) passage seven of Marc145 cells infected with the isolated EV-Gs.


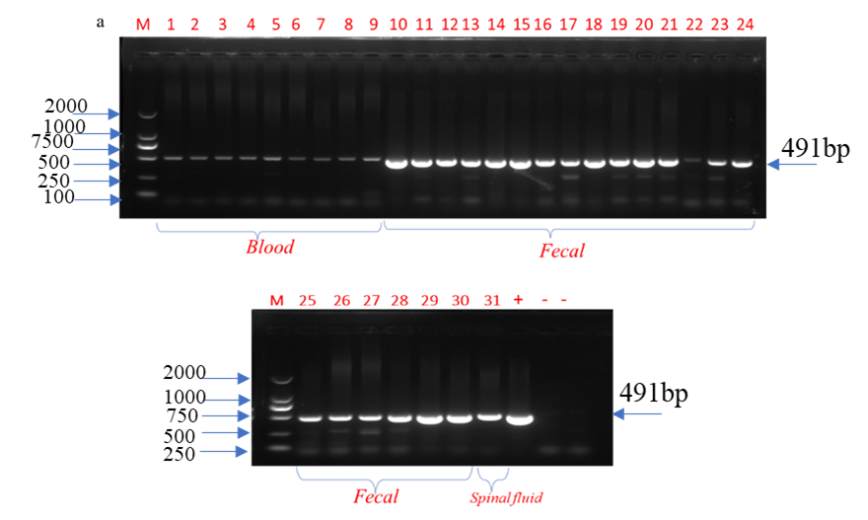


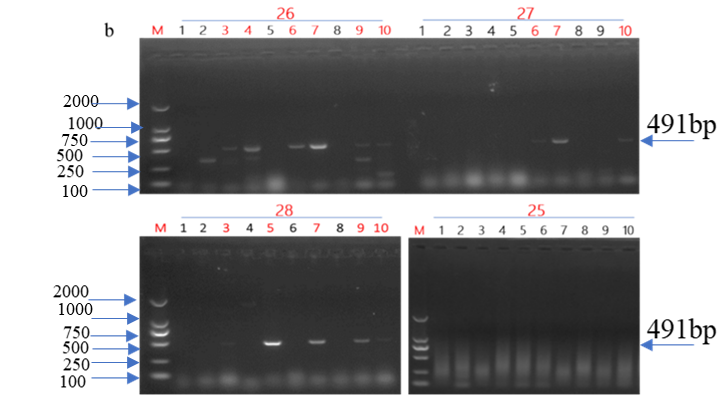


**Fig. S2** Distribution of EV-Gs in blood, feces, spinal fluid, and other tissues of experimental piglets. (a) lanes1-9: blood samples at 3, 5 and 7 dpi, lanes10-30: daily fecal samples from infected animals at day 1 to day 7 post-inoculation, lane 31: spinal fluid from No. 28 piglet**;** (b) Different tissues from EV-G-inoculated animals (No. 26, 27, and 28) and control piglet (No.25). Lane 1: lung, lane 2: liver, lane 3: tonsil, lane 4: pancreas, lane 5: ileum, lane 6: colon, lane 7: rectum, lane 8: mesenteric lymph node, lane 9: cerebrum, lane 10: cerebellum.


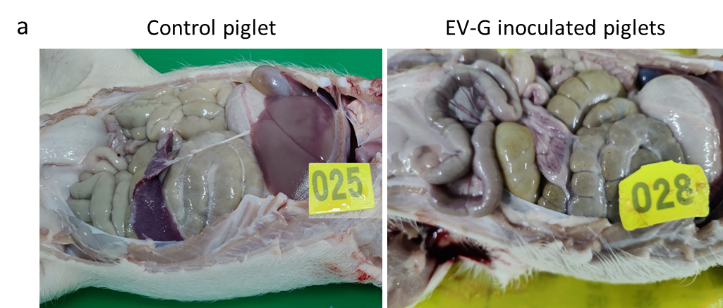

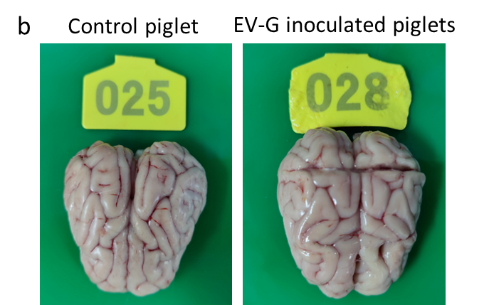


**Fig. S3** Gross anatomy of experimental piglets

**Table S1.** complete genome sequence details of isolated EV-G strains

| Strain |  | Nucleotide and deduced amino acid lengths of region in isolated EV-G | | | | | | | | | | | | | | | | |
| --- | --- | --- | --- | --- | --- | --- | --- | --- | --- | --- | --- | --- | --- | --- | --- | --- | --- | --- |
|  | 5’ UTR  nt | | VP4  nt  (aa) | VP2  nt  (aa) | VP3  nt  (aa) | VP1  nt  (aa) | 2A  nt  (aa) | 2B  nt  (aa) | 2C  nt  (aa) | PLCP  nt  (aa) | 3A  nt  (aa) | 3B  nt  (aa) | 3C  nt  (aa) | 3D  nt  (aa) | ORF  nt  (aa) | 3’ UTR  nt | Complete genome |  |
| CH/HLJ-141 | 813 | | 207  (69) | 738  (246) | 831  (277) | 729  (243) | 450  (150) | 297  (99) | 987  (329) | 639  (213) | 267  (89) | 66  (22) | 549  (183) | 1383  (461) | 7146  (2382) | 71 | 8030 |  |
| CH/HLJ-214 | 813 | | 207  (69) | 738  (246) | 831  (277) | 729  (243) | 450  (150) | 297  (99) | 987  (329) | 639  (213) | 267  (89) | 66  (22) | 549  (183) | 1383  (461) | 7146  (2382) | 71 | 8030 |  |
| CH/HLJ-312 | 813 | | 207  (69) | 738  (246) | 831  (277) | 729  (243) | 450  (150) | 297  (99) | 987  (329) | 639  (213) | 267  (89) | 66  (22) | 549  (183) | 1383  (461) | 7146  (2382) | 71 | 8030 |  |
| CH/HLJ-315 | 813 | | 207  (69) | 738  (246) | 831  (277) | 729  (243) | 450  (150) | 297  (99) | 987  (329) | 639  (213) | 267  (89) | 66  (22) | 549  (183) | 1383  (461) | 7146  (2382) | 71 | 8030 |  |

**Table S2.** Identity comparison of complete genome between isolated EV-G strains

| Strain | Nucleotide and deduced amino acid identities (%) |
| --- | --- |
|  | CH/HLJ-141 CH/HLJ-214 CH/HLJ-312 CH/HLJ-315 |
|  | nt aa nt aa nt aa nt aa |
| CH/HLJ-141  CH/HLJ-214 98.8 99.5  CH/HLJ-312 99.2 99.9 98.8 99.5  CH/HLJ-315 99.2 99.9 98.8 99.5 100 100 | |

**Table S3.** Reference sequence of PEVG strains used in this study. The strain, collected date, country, and accession number of each strain

| **No** | **Strain** | **Host** | **Year** | **Country** | **Accession No.** |
| --- | --- | --- | --- | --- | --- |
| 1 | Chahf1/G1 | Swine | 2008 | China | HM131607 |
| 2 | GXQZ/G1 | Swine | 2017 | China | MT274669 |
| 3 | Iba46431/G1 | Swine | 2015 | Japan | LC316790 |
| 4 | 410/73/G1 | Swine | 2002 | UKG | AF363453 |
| 5 | 410/73/G1 | Swine | 2002 | UKG | Y14459 |
| 6 | 1303212/G1 | Swine | 2013 | USA | KF985175 |
| 7 | KNU1811/G1/PLCP | Swine | 2018 | Korea | MH663501 |
| 8 | Texas1/G1/PLpro | Swine | 2014 | USA | KY498016 |
| 9 | Texas2/G1/PLpro | Swine | 2014 | USA | KY498017 |
| 10 | Iba46441/G1/PLCP | Swine | 2015 | Japan | LC316778 |
| 11 | HgOg23/G1/PLCP | Swine | 2015 | Japan | LC316775 |
| 12 | 5V010/ G1/PLPC | Swine | 2015 | Belgium | KY214435 |
| 13 | LP_54 /G2 | Swine | 2001 | Germany | AF363455 |
| 14 | Iba26506/G2 | Swine | 2014 | Japan | LC316792 |
| 15 | HgYa21/G2/PLCP | Swine | 2015 | Japan | LC316791 |
| 16 | IshiSa5/G3 | Swine | 2015 | Japan | LC316808 |
| 17 | IshiKa7/G3 | Swine | 2016 | Japan | LC316815 |
| 18 | Bu84/G3 | Swine | 2014 | Japan | LC316807 |
| 19 | Bu82/G3 | Swine | 2014 | Japan | LC31680 |
| 20 | Bu65/G3 | Swine | 2014 | Japan | LC316805 |
| 21 | K23/G3 | Swine | 2008 | Hungary | HQ702854 |
| 22 | PoEnVBEL12R021/G3 | Swine | 2015 | Belgium | KP982873 |
| 23 | WBD/G4 | Swine | 2011 | Hungary | JN807387 |
| 24 | HgYa11/G4 | Swine | 2016 | Japan | LC316818 |
| 25 | TB4OEV/G5 | Ovine | 2009 | Hungary | JQ277724 |
| 26 | PEVBKOR/G6 | Swine | 2009 | Korea | JQ818253 |
| 27 | 990/UKN-I/G7 | Swine | 2018 | UKG | MG958646 |
| 28 | 724118/G8 | Swine | 2012 | Vietnam | KJ156437 |
| 29 | 714418/CaoLanh_VN/G8 | Swine | 2012 | Vietnam | KT265911 |
| 30 | IshiYa32/G9 | Swine | 2016 | Japan | LC316825 |
| 31 | Iba2720/G9 | Swine | 2015 | Japan | LC316824 |
| 32 | HgTa222/G9 | Swine | 2015 | Japan | LC316821 |
| 33 | 714152/CaoLanh_VN/G9 | Swine | 2012 | Vietnam | KT265893 |
| 34 | 714171/CaoLanh_VN/G9 | Swine | 2012 | Vietnam | KT265894 |
| 35 | 724162/G9 | Swine | 2012 | Vietnam | KJ156438 |
| 36 | 734087/ThanhBinh/G9 | Swine | 2012 | Vietnam | KT265961 |
| 37 | IshiKa32/G10 | Swine | 2015 | Japan | LC316829 |
| 38 | IshiIm8/G10 | Swine | 2016 | Japan | LC316830 |
| 39 | HgYa241/G10 | Swine | 2015 | Japan | LC316828 |
| 40 | HgYa231/G10 | Swine | 2015 | Japan | LC316827 |
| 41 | 734123/G10 | Swine | 2012 | Vietnam | KJ156446 |
| 42 | 744257/G11 | Swine | 2012 | Vietnam | KJ156451 |
| 43 | 14222/CaoLanh_VN/G12 | Swine | 2012 | Vietnam | KT265900 |
| 44 | 714270/CaoLanh_VN/G13 | Swine | 2012 | Vietnam | KT265903 |
| 45 | 714405/CaoLanh_VN/G14 | Swine | 2012 | Vietnam | KT265909 |
| 46 | 724307/ChauThanh_VN/G15 | Swine | 2012 | Vietnam | KT265941 |
| 47 | BS14173H2/DakLak_VN/G16 | Swine | 2014 | Vietnam | KT266010 |
| 48 | EVG08/G17 | Swine | 2015 | USA | KY761948 |
| 49 | F262/G18 | Swine | 2013 | Germany | MF113370 |
| 50 | F82/G19 | Swine | 2013 | Germany | MF113372 |
| 51 | JL14/G20 | Goat | 2014 | China | KU297674 |
